# Using a hybrid agent-based and equation based model to test school closure policies during a measles outbreak

**DOI:** 10.1186/s12889-021-10513-5

**Published:** 2021-03-12

**Authors:** Elizabeth Hunter, John D. Kelleher

**Affiliations:** Technological University Dublin, Grangegorman, Dublin, Ireland

**Keywords:** Agent-based model, Infectious disease, School closure, Measles, Simulation, Hybrid model, Ireland

## Abstract

**Background:**

In order to be prepared for an infectious disease outbreak it is important to know what interventions will or will not have an impact on reducing the outbreak. While some interventions might have a greater effect in mitigating an outbreak, others might only have a minor effect but all interventions will have a cost in implementation. Estimating the effectiveness of an intervention can be done using computational modelling. In particular, comparing the results of model runs with an intervention in place to control runs where no interventions were used can help to determine what interventions will have the greatest effect on an outbreak.

**Methods:**

To test the effects of a school closure policy on the spread of an infectious disease (in this case measles) we run simulations closing schools based on either the proximity of the town to the initial outbreak or the centrality of the town within the network of towns in the simulation. To do this we use a hybrid model that combines an agent-based model with an equation-based model. In our analysis, we use three measures to compare the effects of different intervention strategies: the total number of model runs leading to an outbreak, the total number of infected agents, and the geographic spread of outbreaks.

**Results:**

Our results show that closing down the schools in the town where an outbreak begins and the town with the highest in degree centrality provides the largest reduction in percent of runs leading to an outbreak as well as a reduction in the geographic spread of the outbreak compared to only closing down the town where the outbreak begins. Although closing down schools in the town with the closest proximity to the town where the outbreak begins also provides a reduction in the chance of an outbreak, we do not find the reduction to be as large as when the schools in the high in degree centrality town are closed.

**Conclusions:**

Thus we believe that focusing on high in degree centrality towns during an outbreak is important in reducing the overall size of an outbreak.

## Background

Infectious disease outbreaks are a major threat to global health while emerging diseases such as COVID-19, SARS, Ebola, and MERS tend to grab the biggest headlines infectious diseases such as influenza, measles and mumps are becoming more prevalent due to a reduction in vaccination rates and global travel. In 2018 there were almost 10 million cases of measles with 142,000 deaths around the world and in 2019 there were three times as many cases reported as in 2018 [1]. As outbreaks get larger, many governments try to impose control measures by closing schools, starting vaccination campaigns, or limiting public events. In order to help mitigate outbreaks and choose the best interventions it is important to understand as much as we can about outbreak dynamics. An intervention is not useful if it takes up resources and does nothing to alter the course of an outbreak or makes an outbreak worse. Additionally an intervention that only has a minor impact on an outbreak but uses a considerable amount of resources might not be the best strategy. However, it is difficult to test if an intervention works during a real outbreak. One main reason for this is there is no control scenario to compare what would have happened if the intervention was not implemented. So it is difficult to determine what was a result of the intervention and what would have happened if the intervention was not implemented.

One way to better understand how interventions influence an outbreak is by using models. Models are often used to study a system when it would be infeasible to run an experiment to study the real world system. Two of the main types of epidemiological models often used to study infectious disease outbreaks are agent-based and equation based models. We use a hybrid agent-based and equation based model to simulate the spread of measles through a county in Ireland. Our motivation for using a hybrid model is that it enables us to scale the model to include a larger population while at the same time controlling the computational cost of running the simulation. We use the model to look at intervention policies in particular school closure policies, we run a number of different scenarios to determine which policy is the best in reducing outbreaks. The paper starts by reviewing the literature for agent-based models used to analyse interventions in particular school closure policies. Then we discuss the model used to look at interventions, and then outline our experiments. Finally, we present the results.

### Modelling the effect of school closures on infectious disease spread

An intervention strategy that is occasionally implemented but its effectiveness is still debated is school closure policies. The policy of closing schools during disease outbreaks is used in Japan, Bulgaria and Russia to lessen influenza outbreaks [2]. Its usefulness was debated in New York during the 1918 Spanish flu pandemic [3] and is being used today in an attempt to reduce the severity of measles outbreaks in Samoa [4] and to reduce the spread of COVID-19 in countries around the world. However, there is no clear evidence to show that closing schools helps to reduce the size of an outbreak. In fact, Lee et al. [5] find that shorter school closures of two weeks or less end up increasing the overall attack rate^1^ and school closures may only be effective if they last for the entire duration of the epidemic. Similarly, Grefenstette et al. [6] find that while the epidemic temporarily slows when schools close as soon as they reopen the epidemic peaks again. However, there are cases where studies have shown that school closure policies can play a significant role in reducing an outbreak. Litvinova et al. [2] use real contact data to simulate the effects of a Russian school closure policy and find that reactive strategies, closing down classes and schools when a given percent of students show symptoms reduces the severity of an outbreak. One of the reasons why the effects of school closure policies is hard to determine is that not all policies are the same. Many factors help to define a school closure policy such as the number of students needed to be infected before implementing the policy, the length of the closure and determining what schools close down. Is it enough to only close the effected schools or should all schools in the town or all schools within a certain radius be closed to reduce the spread of the infectious disease. We aim to use a model to test school closure policies that take into account a towns place in a network of other towns: focusing on the centrality of other towns in the network and the physical distance to other towns.

## Methods

The model used for the study is at its core an agent-based model. The basic building blocks of an epidemiological agent-based model are an environment component, a transport component, a society component and a disease component [7]. We use the model presented in Hunter et al. [8] that was created to model the spread of measles through an Irish county. The code for the model and accompanying documentation including a detailed description of the model (in the Overview, Design concepts and Details protocol, which is a general standard format for describing agent-based models, see [9] for details), sub-models and model schedule is published online and can be found at [10]. The model is a discrete time model with twelve time steps making up a day in the model. Here we give a brief overview of the four components of the model but see [8] for more detailed explanations. The environment component is created using Irish Census data, breaking down the environment into small areas (census areas that contain between 50 to 200 dwellings) [11]. Data from the Irish Department of Education is used to determine the number of primary and secondary schools in each small area. The society component of the model is also created with Irish census data. The distribution of age, sex, economic status and household status for agents in each small areas matches that of the population of the small area. Agents are given a number of social networks that define their family, school and work contacts. Vaccination rates for measles are included in the model and match Irish measles vaccination rates. The transportation component of the model is made up of two parts: students and working agents will move between school, work and home at predetermined times in the model and during non school/work times for students and working agents or daytime for non working agents the agents will move throughout the county using a gravity model that pulls agents towards small areas with high population density and pushes agents away from small areas that are physically farther away. The disease component of the model has both an agent-based component and an equation based component. The disease component for a town (a collection of small areas) will switch from an agent-based to equation-based disease component when 1% of agents are infected or exposed in that town. The theory behind this switch is that the agent-based component is most important when the number infected in the population is low because individual actions will play a larger role in the driving the spread of the disease. If an infected agent comes into contact with a susceptible agent there is a chance that the infected agent will pass on the virus. If they do, the newly infected agent will move between exposed, infected and recovered states. The equation-based component uses a set of difference equation models that can be found in [8]. The disease dynamics of the model are set to mimic measles: an agent will be exposed for an average of 10 days and infectious for an average of 8 days [12]. The infection rate, the percentage chance that a susceptible agent will be infected after contact with an infectious agent, is determined using the basic reproductive number for measles (12-18) [12], based on the method used in [13] we determine the transmission probability per contact to be 0.002.

### Model implementation

For the model implementation we use the county of Leitrim Ireland. We consider the county in isolation and do not include commuting to or from other counties. From the 2016 census the county has a population of approximately 36,000 people over an area of 1,590km^2^ and is made up of 173 small areas and 69 different electoral divisions (for the purpose of this study we consider an electoral division as equivalent to a town). About 45% of the population are students and using Irish vaccination rates, 12.3% of the population is not vaccinated or otherwise immune to measles, this equates to a susceptible population of 3,936. There are 7 secondary schools and 37 primary schools in Leitrim in 31 different towns. In our experiments outlined in the next section we consider different school closure policies on 9 of the towns in Leitrim. These 9 towns were chosen because either they had the highest in degree centrality in the commuting network between the towns within the county or their close physical distance to the starting location of the outbreak, and they had a population greater than 450 individuals. The selection of the 9 towns is further discussed in Experiments section. A summary of the characteristics of the 9 towns including total population, the number of primary and secondary schools, the number of students who live in the town, the total number of students that attend school in the town (this includes students who commute in) and the number of agents who are not immune to measles can be found in Table 1. Note that the final row in the table lists the statistics for the full county, also there is a town in the county of Leitrim that is also called Leitrim. Furthermore, while we focus our analysis of the results on the 9 towns where close schools, it is important to note that the model includes all towns and agents can commute between any of the 69 towns in the county.

**Table 1:** Town Characteristics for the nine towns in Leitrim that are considered in the school closure policies

Town	Population	Primary	Secondary	Students	Total Students	Susceptible
Ballinamore	1,265	2	1	576	1,685	151
Carrigallen	956	1	1	419	1,356	137
Drumahaire	1,640	2	0	713	241	185
Drumkeeran	528	1	1	243	1,367	77
Drumshanbo	1,631	1	1	778	1,738	205
Killanummery	456	1	0	222	126	57
Leitrim (town)	1,319	1	0	595	270	154
Manorhamilton	2,059	3	1	875	2,028	248
Mohill	1,357	2	1	564	2,006	158
Leitrim County	36,551	37	7	16,673	16,673	4,655

Characteristics include the total population of the town, the number of primary schools, the number of secondary schools, the number of students who live in the town, the total number of students who attend school in the town including those commuting in and the number of agents in the town who are not immune and thus susceptible to measles

**Fig. 1:**
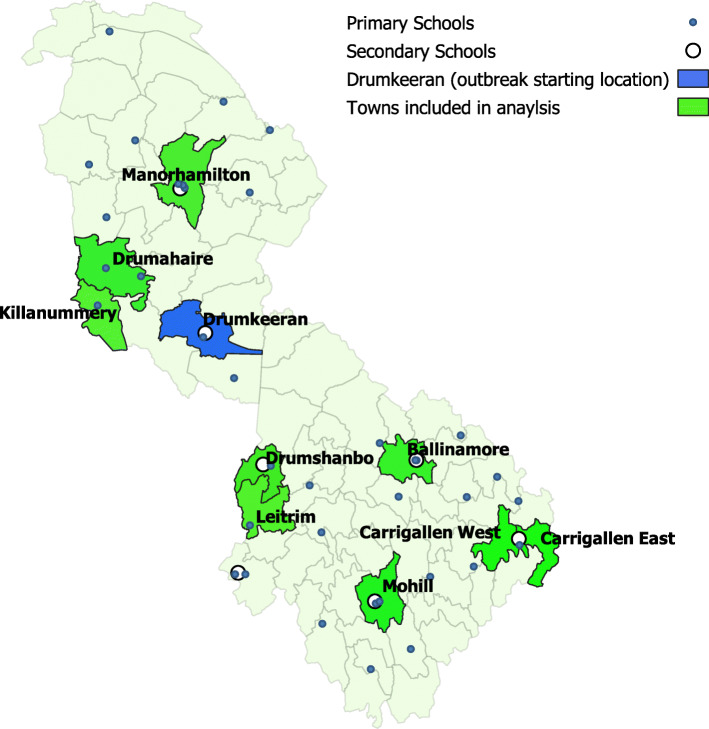
Map of Leitrim County showing locations of primary and secondary schools. The towns included in the analysis are darker green. Created using QGIS [14] using openly available Irish Census data [11] and Irish Department of Education data [15]

Figure 1 shows a map of Leitrim county. Each electoral division (which we equate to a town) is outlined in the map with the towns used in our analysis shaded in a darker green. The locations of all primary and secondary schools are included and the town where the outbreaks in our experiments described in the following sections begin is highlighted in blue. Although we use Leitrim as an example in this paper, any county in Ireland can be used for the model or any region in another country as long as we have the appropriate data.

The model will start with one agent infected and will run until there are no longer any agents infected or exposed.

### Experiments

**Fig. 2:**
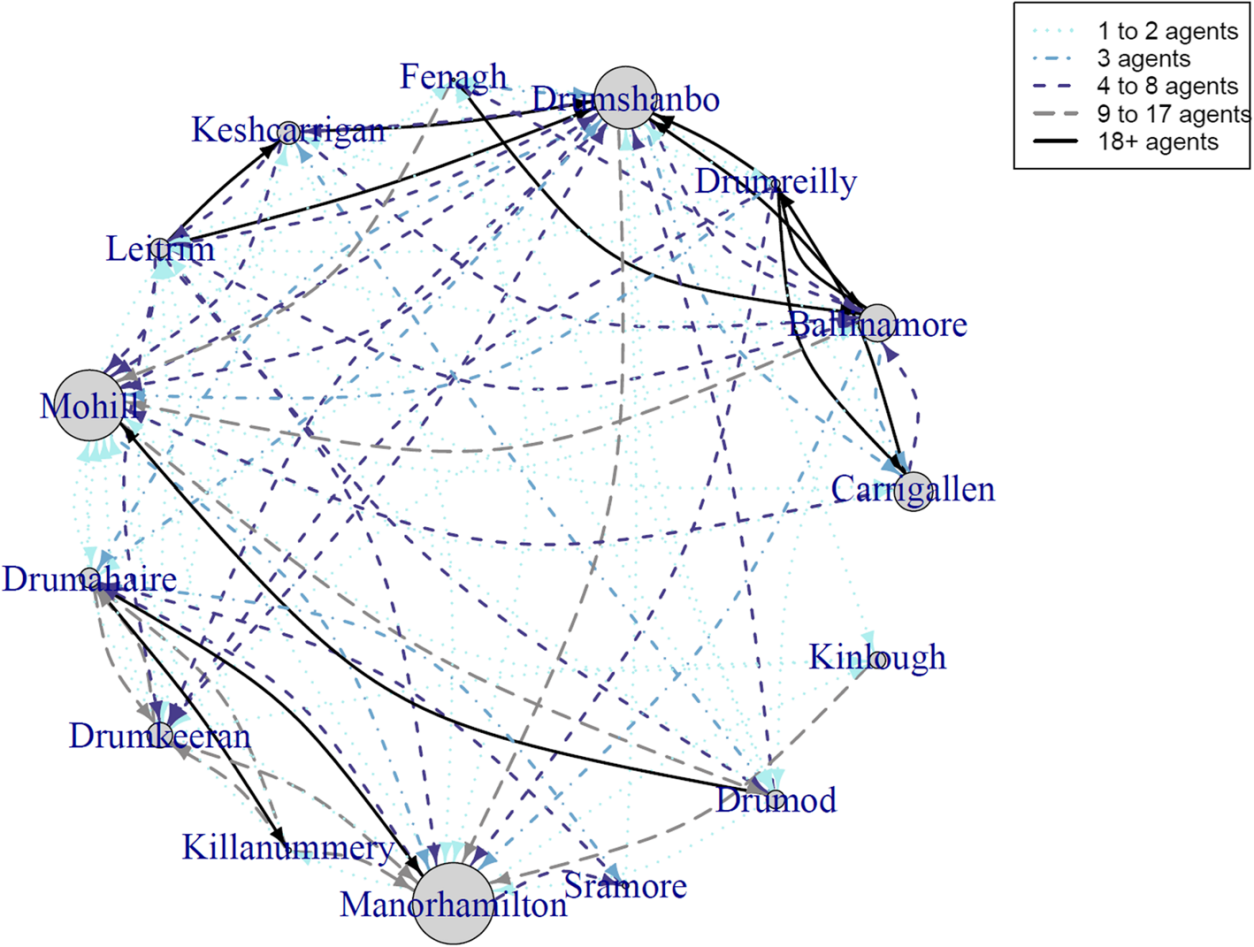
Graph showing the commuting patterns between the towns in Leitrim that have a population greater than 450 and have a primary or secondary school. The colors of the edges of the graph represent the number of agents commuting along the link and the size of the node relates to the centrality of the town. The larger the node the greater centrality

Having a model that can recreate an outbreak allows us to learn interesting things about the dynamics of an infectious disease outbreak, for example Hunter et al. [16] examines how the centrality of a town within a network of towns influences the spread of an outbreak. In the study the degree centrality of towns in a network is considered and is determined based on the number of agents commuting into and out of a town. There are multiple types of degree centrality: *total degree centrality* weights agents commuting in and out of towns equally, *in degree centrality* is based on the count of agents commuting into a town, and *out degree centrality* is based on the count of agents commuting out of a town. They find that the agents commuting into a town are more important in spreading an infectious disease than the agents commuting out of a town and that the higher the in degree centrality of the town the outbreak starts in the less important the centrality of the other towns in the network is in determining if the outbreak will spread to those other towns. However, even though this is an interesting finding, the question remains how can this help us in stopping or slowing down an outbreak. We propose using the findings from the Hunter et al. [16] model to test out different intervention strategies. For example, while it might seem to make sense to close the schools in a town when an outbreak of a childhood disease begins to take off, there is evidence to show that this does not always help and in some cases actually makes an outbreak worse. As it was determined that a town with higher in degree centrality will result in greater spreading of the outbreak across all towns in the network, we run experiments to look at the effects of closing down schools in the high in degree centrality towns as opposed to the town the outbreak starts in. The thought behind this is that it is the high in degree centrality towns that results in spreading to more towns throughout the network and that by stopping agents students from going into these high centrality towns we will stop them from bringing the disease into the high centrality town and then out to other towns. Fig. 2 shows the commuting network between the towns in Leitrim that have at least one primary or secondary school and have a population greater than 450. The colors of the edges of the graph represent the number of agents commuting along the link. The lightest shade of blue is the first quartile of agents commuting, the gray edges are the fourth quartile of agents commuting between towns and the black edges are commuting connections between towns where the number of agents commuting are large outliers. This color based encoding of the amount of agents commuting along an edge is supplemented by the line type encoding, with a dotted line indicating the first quartile of agents commuting, and a full line indicating that the number of agents commuting along the edge is so large as to be considered an outlier. Edges are directional with the arrow pointed in the direction of the commute. Two towns could have two separate edges connecting if there is commuting in both directions. The centrality of each town is encoded by the size of the node representing the town. The larger the node the greater the centrality. It is important to note that the graph only represents the commuting patterns between 15 of the towns in the county but the centrality is based off of commuting between all towns. From the graph we can see that while some towns with schools, such as Manorhamilton or Mohill, are highly central and have many links commuting in and out of the town, other towns such as Kinlough or Sramore have far fewer links compared with other towns in the network.

In order to test the effects of closing schools in different towns we run two experiments using the hybrid model. The first experiment involves four different intervention scenarios. The first scenario is with no interventions but vaccination rates based off of Irish vaccination rates and with the outbreak starting in the town of Drumkeeran, Ireland in County Leitrim. Drumkeeran was chosen as it is a smaller town in Leitrim County with relatively low in degree centrality and it has both a primary and a secondary school in the town. The second intervention scenario involves running the hybrid model with vaccination rates and with schools in Drumkeeran closing down when more than two students are infected in the town. The third scenario again uses vaccinations and when more than two students are infected in Drumkeeran, the schools in Manorhamilton are shut down. Manorhamilton is the town in Leitrim with the highest in degree centrality and is approximately 20 km away from Drumkeeran. The final intervention scenario has the same vaccination rates and involves closing schools down in both Drumkeeran and Manorhamilton when more than two students are infected. For each intervention scenario the model is run 300 times to account for stochasticity [17] and we look at a number of different statistics to compare the scenarios. We look at three different measures for comparison: the total number of runs that lead to an outbreak out of the 300 runs, the total number of infected agents, and the geographic spread of the outbreak.

The second experiment involves looking at different combinations of school closures based on the centrality of the towns versus the distance of the town to the initial outbreak. The outbreak begins in Drumkeeran for each set of runs. The model was run to test each of the following intervention scenarios based on in degree centrality: (i) only closing the schools in Drumkeeran; (ii) closing the schools in Drumkeeran and the schools in the town with the highest in degree centrality that has at least one primary or secondary school, this second town was Manorhamilton (see Table 2 for a listing of the five town in Leitrim containing at least one school ordered by in degree centrality); (iii) closing the schools in Drumkeeran and the schools in the town with the highest in degree centrality (i.e, Manorhamilton) and the town with the second highest in degree centrality (i.e., Mohill); and so on, with runs (iv), (v), and (vi) each progressively adding the towns included in the school closures in order of decreasing in degree centrality.

**Table 2:** Towns in Leitrim with at least one school ordered by centrality

Town	Centrality
Manorhamilton	197.8
Mohill	171.7
Drumshanbo	151.8
Carrigallen	93.7
Ballinamore	90.8

In this second experiment, the model was also run to test school closure interventions based on distance from the initial infection. This is done to help determine if any results from closing down schools by centrality is simply because additional schools are closed or if it can be attributed to the centrality of the town that is closed. We calculate the distance between towns as the distance between the centroids of each town. The first run closes school in only Drumkeeran, the town where the outbreak starts; the next set of runs closes schools in Drumkeeran and the next closest town with at least one school in it; the third set of runs closes schools in Drumkeeran, the closest town with at least one school in it, and the second closest town with at least one school; and so on. Table 3 shows a list of the five closest towns to Drumkeeran.

**Table 3:** Towns in Leitrim with at least one school ordered by distance to Drumkeeran

Town	Distance (km)
Drumahaire	14
Killanummery	14
Drumshanbo	18
Manorhamillton	20
Leitrim (town)	20

For each set of towns the model is run 300 times and the results are compared between closing schools based on distance and centrality. Following the analysis methods used in experiment one, in this second experiment we use the same three measures to compare the interventions: the total number of runs that lead to an outbreak out of the 300 runs, the total number of infected agents, and the geographic spread of the outbreak.

## Results

We first look at the results for the first experiment: vaccination^2^, closing schools in Drumkeeran^3^, closing schools in Manorhamilton^4^ and closing schools in Drumkeeran and Manorhamilton^5^. The first measure we look at to compare the results of the different interventions is to look at the percent of runs that lead to more than three agents infected. In a model without interventions we typically look at the percent of runs that lead to an outbreak, using the World Health Organization’s definition of a measles outbreak which is two or more connected cases of measles, however, because we want to look at the effects of the interventions, and the interventions do not start until we have at least two agents infected, we look at the runs where there are more than three agents infected. Table 4 shows the percent of runs that have three or more infected agents for each of the versions of the model along with the 95% confidence intervals for the statistics and the p-value for a test of equal proportions to show if the percent of runs resulting in three or more agents being infected is significantly different from the percent of runs resulting in three or more agents being infected in the vaccinations only model.

**Table 4:** The percent of runs that result in three or more agents becoming infected based off of the intervention strategies used in the model

Intervention	Percent of Runs	Confidence Interval	*P*-value
Vaccinations Only	51.3	(45.7, 57.0)	-
Drumkeeran	51.3	(45.7, 57.0)	1.00
Manorhamilton	47.3	(41.7, 53.0)	0.37
Drumkeeran and Manorhamilton	43.3	(37.8, 48.9)	0.06

The 95% confidence interval and the *p*-value for a test of equal proportions. The first intervention is vaccinations with no school closures and the next three rows give results for the schools that are closed in the simulation to stop the spread of measles

From Table 4 it can be seen that the percent of runs that lead to three or more infected agents is the same for the model with only vaccination as an intervention and the model closing schools in Drumkeeran. This further emphasises the findings showing that closing schools in the town where the outbreak starts does not always reduce an outbreak. Looking at the effect of the other interventions on the model results we can see that even though the percent of runs with over three infected when schools are closed in Manorhamilton is slightly lower than the percent of runs when the schools are closed in Drumkeeran the results are not significantly different. However, when schools close in both Drumkeeran and Manorhamilton, the percent of runs with more than three infected agents is significantly different at a 7% significance level than when vaccinations are the only intervention or when only schools in Drumkeeran are closed down. These results show that there might be an advantage in closing down the schools in the high centrality towns nearby along with the initial town where the outbreak starts.

The results are further broken down to see if there are other effects of closing schools. We look at some summary statistics for the total number of infected agents at the end of the outbreak for the county wide outbreak for all versions of the model in Table 5.

**Table 5:** Summary statistics for the number of infected agents across model runs where at least 3 agents were infected by intervention, including the confidence interval for the mean

Intervention	1st Quartile	Median	Mean	3rd Quartile	Max
Vaccinations Only	15	38	48.71	64	492
			*(40.14, 57.27)*		
Drumkeeran	15	38	78.09	74	4253
			*(26.97, 129.21)*		
Manorhamilton	13	38	48.06	66	652
			*(37.89, 58.22)*		
Drumkeeran and Manorhamilton	15	41	47.94	71	210
			*(41.37, 54.5)*		

The first intervention is vaccinations with no school closures and the next three rows give results for the schools that are closed in the simulation to stop the spread of measles

From Table 5 we can see that there are some distinct differences between the results across the different interventions. In particular when looking at the mean value across the runs the mean number of infected agents is lower when schools are closed in Manorhamilton and when schools are closed in both Drumkeeran and Manorhamilton compared to schools closed in only Drumkeeran. However, the maximum value for the total number of infected agents across the 300 runs is much higher for when the model runs with schools closing in Drumkeeran versus when schools close in Manorhamilton or both Drumkeeran and Manorhamilton. This extremely high value, 4,235, is an outlier in the Drumkeeran school closure runs and is what is driving the mean of those runs up. Looking at the medians, a statistic that is resistant to outliers, we can see that there is almost no difference between the runs.

Additionally we look at how the outbreak spreads beyond the initial town within the network. The first measure that we look at is the number of cases of the disease outside of the initial town. Table 6 shows the percent of runs that lead to an outbreak (two or more infected) anywhere in the model and the percent of runs that have at least one agent infected outside of Drumkeeran. The results show that for all intervention strategies the disease does not spread outside of Drumkeeran on every run. The p-values reported in Table 6 were calculated by comparing the outcomes of each of the school closure intervention strategies to the vaccination only strategy in terms of the number of runs when at least one agent outside of Drumkeeran is infected. From the results we can see that there is no statistical difference between the runs when there is at least one agent infected outside of Drumkeeran for the model with only vaccination, the model where schools are closed in Drumkeeran or the model where schools are closed in Manorhamilton. However, we do see a smaller result for the model where schools are closed in both Drumkeeran and Manorhamilton that is significant at the 10% level.

**Table 6:** A comparison between the percent of runs that lead to an outbreak (2 or more infected agents) and the percent of runs where at least one agent is infected from outside of Drumkeeran the initial town and the 95% confidence intervals for those and the *P*-value comparing the results to the vaccination only model

Intervention	Outbreak	Outside Drumkeeran	*P*-value
Vaccinations Only	63.3	49.7	-
	*(57.9, 68.8)*	*(44.0, 55.3)*	
Drumkeeran	62.0	50.3	0.95
	*(56.5, 67.5)*	*(44.7, 56.0)*	
Manorhamilton	61.7	47.3	0.61
	*(56.2, 67.2)*	*(41.7, 53.0)*	
Drumkeeran and Manorhamilton	57.0	42.7	0.10
	*(51.4,62.6)*	*(37.1, 48.3)*	

Although when comparing the scenarios we do not see a difference in the total number of agents infected, when we look at both the total number of runs that lead to an outbreak after schools have closed and the geographic spread (in terms of the number of runs where there are infections outside of Drumkeeran) there is a reduction in the number of runs where an outbreak will occur when schools are closed in both Drumkeeran and Manorhamilton and a reduction in the number of runs where the outbreak spreads outside of Drumkeeran.

In the second experiment we look at closing schools based on their in degree centrality. Schools initially close down in the town that the outbreak starts, Drumkeeran, then Drumkeeran and the town with the next highest centrality, Manorhamilton, then Drumkeeran, Manorhamilton and the town with the next highest centrality, Mohill. We look at the percent of runs where three or more agents are infected. The results for this experiment are found in Table 7. From the table we can see that when we close schools in two towns (Drumkeeran and Manorhamilton) there is a drop in the percent of runs that lead to an outbreak that is statistically significant at the 7% level but that when the school closure intervention is extended to more towns the percent rises and is no longer significantly different from the vaccination only model or the model where only schools in Drumkeeran are closed. This is an interesting finding but may be due to the fact that the students whose schools are closed do not change their behaviour in response to the outbreak. Instead of going to school they will treat the days off as if it were a weekend and thus will interact with each other potentially spreading the disease if an infected student decides to leave their home. For this network of towns and population distribution, closing schools in three towns may be the tipping point from which closing schools reduces the outbreak to a situation where closing schools does not have an effect.

**Table 7:** The percent of runs that result in three or more agents becoming infected when schools are closed by in degree centrality

Additional Towns Closed	Percent of Runs	Confidence Interval	*P*-Value
Vaccinations Only	51.3	(45.7, 57.0)	-
Drumkeeran	51.3	(45.7, 57.0)	1.00
Manorhamilton	43.3	(37.8, 48.9)	0.06
Mohill	47.3	(41.7, 53.0)	0.37
Drumshanbo	51.3	(45.7, 57.0)	1.00
Carrigallen	49.0	(43.3, 54.7)	0.62
Ballinamore	54.0	(48.4, 59.6)	0.57

We also look at the summary statistics for the runs that lead to at least three agents infected for school closures based on centrality, these can be found in Table 8. Looking at the table it is clear that for the runs where an outbreak does occur, there is limited difference between the outbreaks based on where schools have closed.

**Table 8:** Summary statistics for the number of infected agents across model runs when closing schools by centrality where at least 3 agents were infected by the intervention, including the confidence interval for the mean

Additional Town Closed	1st Quartile	Median	Mean	3rd Quartile	Max
Drumkeeran	15	38	78.09	74	4253
			*(26.97, 129.21)*		
Manorhamilton	13	38	48.06	66	652
			*(37.89, 58.22)*		
Mohill	13	38	47.18	68	263
			*(40.5, 53.8)*		
Drumshanbo	15	38	78.83	61	4386
			*(21.3, 126.4)*		
Carrigallen	11	32	73.06	60	4505
			*(16.4, 129.7)*		
Ballinamore	16	38	49.96	67	267
			*(40.5, 53.4)*		

The first row is for schools closed in Drumkeeran, the second is for schools closed in Drumkeeran and Manorhamilton, the third is for schools closed in Drumkeernan, Manorhamilton and Mohill and so forth

To look at how the outbreak spreads beyond the initial town within the network Table 9 shows both the percent of runs that lead to an outbreak (two or more infected) anywhere in the model and the percent of runs that have at least one agent infected outside of Drumkeeran. The p-values reported in Table 9 are comparing the percent of runs outside of Drumkeeran for each of the intervention scenarios to the percent of runs outside of Drumkeeran in the vaccination only scenario with no school closures. Based on the results we can see that the difference between the runs when there is at least one agent infected outside of Drumkeeran for the model with only vaccination and the model where schools are closed in both Drumkeeran and Manorhamilton is statistically significant at the 10% level. Thus showing that closing the schools in both towns reduces the geographic spread of the outbreak.

**Table 9:** A comparison between the percent of runs that lead to an outbreak (2 or more infected agents) and the percent of runs where at least one agent is infected from outside of Drumkeeran when schools are closed based on centrality and the 95% confidence intervals for those and the *P*-value comparing the results to the vaccination only model

Intervention	Outbreak	Outside Drumkeeran	*P*-value
Vaccinations Only	63.3	49.7	-
	*(57.9, 68.8)*	*(44.0, 55.3)*	
Drumkeeran	62.0	50.3	0.95
	*(56.5, 67.5)*	*(44.7, 56.0)*	
Manorhamilton	57.0	42.7	0.10
	*(51.4,62.6)*	*(37.1, 48.3)*	
Mohill	61.0	46.3	0.46
	*(55.5,66.5)*	*(40.7, 52.0)*	
Drumshanbo	62.0	50.3	0.93
	*(56.5,67.5)*	*(44.7, 56.0)*	
Carrigallen	57.3	47.7	0.68
	*(51.7,62.9)*	*(42.0, 53.3)*	
Ballinamore	62.3	52.3	0.57
	*(56.9,67.8)*	*(46.7, 58.0)*	

To determine if the results we found have to do with the centrality of the town and not just the number of towns that the schools are closed in we also look at closing schools progressively by distance to the town where the outbreak starts. Table 10 shows the results for the percent of runs that lead to three or more infections closing towns by distance. The results show that similar to when we close schools by centrality, there is an initial decrease in the percent of runs that have at least three agents infected but this is not as statistically significant as the drop when schools are closed based on centrality (the drop is not significant at the 10% level). This leads us to the conclusion that closing a second town might have a beneficial affect on reducing an outbreak but that closing schools in a town with high in degree centrality is more beneficial than closing schools in a town with the closest distance.

**Table 10:** The percent of runs that result in three or more agents becoming infected when schools are closed in towns by distance

Additional Towns Closed	Percent of Runs	Confidence Interval	*P*-Value
Vaccinations Only	51.3	(45.7, 57.0)	-
Drumkeeran	51.3	(45.7, 57.0)	1.00
Drumahaire	45.3	(39.7, 51.0)	0.16
Killanummery	46.3	(40.7, 52.0)	0.25
Drumshanbo	49.3	(43.7, 55.0)	0.68
Manorhamilton	49.0	(43.3, 54.7)	0.62
Leitrim (town)	46.0	(40.4, 51.6)	0.22

The summary statistics for the runs that lead to at least three agents infected for school closures based on distance can be found in Table 11. Looking at the table it is again clear that for the runs where an outbreak does occur, there is limited difference between the outbreaks based on where schools have closed.

**Table 11:** Summary statistics for the number of infected agents across model runs when closing schools by distance where at least 3 agents were infected by the intervention, including the confidence interval for the mean

Additional Town Closed	1st Quartile	Median	Mean	3rd Quartile	Max
Drumkeeran	15	38	78.09	74	4253
			*(26.97, 129.21)*		
Drumahaire	11	34	70.96	65	3,985
			*(18.20, 123.72)*		
Killanummery	12	42	47.09	69.5	195
			*(40.86, 53.32)*		
Drumshanbo	19	42	49.75	70.75	184
			*(43.64, 55.86)*		
Manorhamilton	14	39	44.55	63	162
			*(38.60, 50.49)*		
Ballinamore	13	35	71.72	61	4,431
			*(13.20,130.23)*		

The first row is for schools closed in Drumkeeran, the second is for schools closed in Drumkeeran and Drumahaire, the third is for schools closed in Drumkeernan, Drumahaire and Killanummery and so forth

Table 12 shows both the percent of runs that lead to an outbreak (two or more infected) anywhere in the model and the percent of runs that have at least one agent infected outside of Drumkeeran for the intervention scenarios where schools are closed based on distance to Drumkeeran, with the p-values comparing the percent of runs outside of Drumkeeran for the intervention scenarios to the percent of runs outside of Drumkeeran in the vaccination only scenario. From the table we can see that there are no statistically significant differences between the runs when there is at least one agent infected outside of Drumkeeran for the model with only vaccination and the models where schools are closed based on distance. However, although not statistically significant at the 10% level we do see a reduction in the number of runs where measles occurs outside of Drumkeeran compared to the vaccination only scenario when schools are closed in Drumkeeran and Drumahaire and smaller reductions when schools are closed in Drumkeeran, Drumahaire and Killanummery and in the scenario when schools are closed in all six towns.

**Table 12:** A comparison between the percent of runs that lead to an outbreak (2 or more infected agents) and the percent of runs where at least one agent is infected from outside of Drumkeeran when schools are closed based on distance to the initial town and the 95% confidence intervals for those and the *P*-value comparing the results to the vaccination only model

Intervention	Outbreak	Outside Drumkeeran	*P*-value
Vaccinations Only	63.3	49.7	-
	*(57.9, 68.8)*	*(44.0, 55.3)*	
Drumkeeran	62.0	50.3	0.95
	*(56.5, 67.5)*	*(44.7, 56.0)*	
Drumahaire	58.3	43.7	0.16
	*(52.8,63.9)*	*(38.0, 49.3)*	
Killanummery	54.7	45.6	0.37
	*(49.0,60.3)*	*(40.0, 51.3)*	
Drumshanbo	63.7	48.7	0.87
	*(56.5,67.5)*	*(43.0, 54.3)*	
Manorhamilton	64.0	48.7	0.87
	*(58.6,69.4)*	*(43.0, 54.3)*	
Leitrim (town)	56	44.3	0.22
	*(50.4,61.6)*	*(38.7, 50.0)*	

## Discussion

Although the literature is undecided about the usefulness of school closure policies on lessening the severity of an outbreak, it is still a commonly used strategy. In this paper we tested different school closure strategies: closing schools in town with high in degree centrality and towns close in distance to the initial town. We found that there was a reduction in the outbreak when closing schools in the initial town and a second town but that selecting the second town using in degree centrality resulted in a larger reduction than selecting the second town using distance. This is likely because the high in degree centrality makes it more likely for an agent to commute to a town with higher centrality bringing the disease into the town. With the schools closed other agents will not become infected at school and bring the disease back to their own town thus reducing the spread. However, as we close schools in more towns, whether this decision is based on centrality or distance, we see that after a certain number of towns have their schools closed then closing schools in more towns actually reduces the overall reduction in outbreaks that the school closure policy achieves when only two towns have closed schools (i.e., resulting in a similar percent of runs leading to an outbreak as only closing schools in Drumkeeran or just vaccinating and closing no schools). This is likely because of an effect that is often cited as reason to not use a school closure policy, that the uninfected and asymptomatic students will still interact just outside of school and will still spread the disease. Introducing some level of restricted movement or self isolation for students who are not in school could lead to different results and greater reductions in outbreaks.

Interestingly, the model results show that while there is a reduction in the total number of runs that lead to an outbreak in some combinations of school closures, for the runs when the outbreak does take off there is almost no difference in the sizes of the outbreak across strategies. This is potentially because of the momentum of an outbreak. In many cases when a second case has occurred there are likely to be other agents who are already exposed but not yet showing symptoms and thus the outbreak will continue to grow in size even if schools are closed. Additionally, as a large number of agents commute into Drumkeeran for school, as can be seen in Table 1, if one of the students commuting into the town are the second infection they will bring measles back to their own town potentially spreading it to siblings who attend a school in another town. Thus for a measles outbreak, school closure may be considered to reduce the chances of a larger outbreak but not to reduce the size of the resulting outbreak.

From the results we can also see that closing schools in the town where the outbreak starts, Drumkeeran, has little effect on stopping the outbreak. This is also likely due to a number of factors including those discussed previously. While the schools are closed there are already at least two agents infected in the town and potentially others who are exposed meaning that the outbreak already has momentum. Also, there is a large number of students commuting into Drumkeeran from other towns, if one of the commuting students is the second case then they are likely to take the disease back to their home and potentially infect family members who may bring the disease to another school leading to a continued outbreak. Another possible factor is that students do not reduce their movements when schools are closed, instead they treat the days when school are closed as they would a weekend or summer holidays. Therefore, infected students who are not staying at home might infect other students from their own town or school while interacting outside of school. However, we do see a reduction in the number of runs where the outbreak spreads outside of Drumkeeran in a number of intervention scenarios showing that the school closures do have an impact on the geographic spread of the outbreak.

Our findings show that closing down the schools in the town where an outbreak begins might not have as much of an effect on reducing the outbreak unless schools in another town are also closed: in particular the schools in towns with the highest in degree centrality will result in the greatest decrease in the potential outbreaks. These findings are the first step in developing intervention strategies to reduce outbreaks based off of town centrality. Additional work can be done to look at the results for different counties. Showing that our results work for Leitrim is one thing but running the same tests for other counties in Ireland or regions in other countries will show that the findings are robust and could be applied anywhere. We could also look at different thresholds for closing down schools: instead of closing down when two agents are infected we could wait for a larger number of students to be infected before the schools close down to determine if this threshold has an impact on the results and which intervention strategies work the best. Additionally, an analysis of school closure policies based on time could be considered, determining if the length of time a school is closed will lead to different outbreak results. There is also the potential to look into changing the agents behaviours after schools close. Instead of moving as if it is the weekend agents could adjust their actions to prevent transmission knowing that there is an outbreak occurring. Similarly, in the current version of the model only students actions are changed when schools close down but the actions of adults whose children attend schools that closed down could also be adjusted. With such adjustment it might also be possible to calculate the economic impact of closing schools down versus letting an outbreak run its course without interventions. This could include the cost of paying teachers salaries while the school is closed and the cost for parents taking the days off from work compared to the cost of treating the number of agents who would be infected. This type of economic analysis is important because although closing down schools in two towns seems to have a beneficial affect on reducing an outbreak, in order to adopt such a policy it would need to be shown that the reduction in the outbreak was not outweighed by the cost of closing down the schools.

Further the work can be extended to show how school closure policies and intervention policies in general might differ based on the disease. While our work focuses on measles, it is important to be able to have a model that can not only simulate the spread of a specific disease such as measles and the interventions for that disease but have a model that can be adapted to other infectious diseases and other interventions. For example, there might be other factors to consider with an emerging disease such as COVID-19 where there is not already a level of immunity in the population. While school closure polices for an infectious disease such as measles might be set into place in order to stop the spread of the outbreak completely, school closure policies for COVID-19 might be put into place to slow the spread of the outbreak and by reducing the number of social contacts each individual has. Although the final number of infected in this case might not be reduced, if the outbreak happens over a longer period of time the maximum number infected at any one time is reduced leading to less of a strain on the health care system. In order to evaluate the ability of an intervention to flatten the outbreak curve, different measures would need to be looked at such as the maximum number of infected agents or the length of the outbreak. Additionally, for a disease such as COVID-19 there is not a pre-existing level of immunity in the population as there is with measles, thus strategies to control the outbreak might differ in other ways for example focusing on workplaces, and other areas in the community where people would gather such as large gyms.

## Conclusion

We aimed to test interventions that look into stopping the outbreak from spreading out of the town of the initial case by looking at the schools in towns that have high levels of in degree centrality, and schools that are close in distance to the initial town and found that we were able to reduce the severity of the outbreak spreading from Drumkeeran when we closed schools not only in the town where the outbreak begins but also in the town with the highest in degree centrality and the towns closest to Drumkeeran. From our finding we determined that closing schools in the town where the outbreak begins and then closing a second town is better at reducing the outbreak than just closing schools in the town where the outbreak initially occurs. In addition, we find that when selecting a second town in which to close the schools, it is more beneficial to choose the town in the region with the highest in degree centrality than it is to choose the nearest town.

These findings should be understood as being grounded in a scenario involving a particular disease (measles) and a particular environment, society, and transport ecosystem (a network of towns in a rural region). We believe that our results do support the general claim that the centrality of a town within a transport network, and in particular the in degree centrality of a town, is an important factor that should be considered when designing an intervention strategy for infectious diseases. However, modelling the details of how in degree centrality affects the dynamics of outbreaks involving other diseases or other contexts would require further modelling experimentation. Indeed, this type of experimentation is one of the strengths of agent-based models as they can be tailored both in terms of the disease and the characteristics of the region and population that is being studied.

## Data Availability

All data sets used to create the model are publicly available. The data from the model output and analysed during the current study are available from the corresponding author on request. The model and model documentation can be found online at [10]

## Footnotes

1 Attack rate is defined as the risk of getting a disease during a specified period.

2 In the vaccination only model, across all runs where an outbreak occurs, on average the disease component in at least one town switched from agent-based to equation based for an average of 8 time steps. The minimum number of time steps that at least one town in the model switched from agent-based to equation based was 0 and the maximum number of time steps was 334.

3 In the model with only schools in Drumeerkan closed, across all runs where an outbreak occurs, on average the disease component in at least one town switched from agent-based to equation based for an average of 16 time steps. The minimum number of time steps that at least one town in the model switched from agent-based to equation based was 0 and the maximum number of time steps was 574.

4 In the model with only schools in Manorhamilton closed, across all runs where an outbreak occurs, on average the disease component in at least one town switched from agent-based to equation based for an average of 9 time steps. The minimum number of time steps that at least one town in the model switched from agent-based to equation based was 0 and the maximum number of time steps was 351.

5 In the model with schools in Drumeerkan and Manorhamilton closed, across all runs where an outbreak occurs, on average the disease component in at least one town switched from agent-based to equation based for an average of 8 time steps. The minimum number of time steps that at least one town in the model switched from agent-based to equation based was 0 and the maximum number of time steps was 195.
